# Microbiological Testing of Gastric Aspirate Improves the Diagnosis of Pulmonary Tuberculosis in Unconscious Adults with TB Meningitis

**DOI:** 10.3390/idr12030025

**Published:** 2020-12-20

**Authors:** Ahmad Rizal Ganiem, Lilya Wati Djung, Lidya Chaidir, Uni Gamayani

**Affiliations:** 1 Department of Neurology, Faculty of Medicine, Universitas Padjadjaran/Hasan Sadikin Hospital, Bandung 40161, Indonesia; lilyawati.djung@gmail.com (L.W.D.); gamayani@yahoo.com (U.G.); 2 Department of Biomedical Sciences, Faculty of Medicine, Universitas Padjadjaran, Bandung 40161, Indonesia; lidya.chaidir@unpad.ac.id

**Keywords:** gastric aspirate, improving diagnosis, pulmonary tuberculosis, tuberculous meningitis

## Abstract

Conventional sputum collection for TB diagnosis is difficult in TB meningitis patients since most of them are admitted with decreased consciousness. It is assumed that unconscious patients swallow their sputum; therefore, gastric aspiration can replace sputum collection in unconscious patients. A prospective study was conducted to see whether examining gastric aspirate could increase the diagnosis certainty of pulmonary TB in such subjects. The inclusion criteria were age 18–60 years, decreased level of consciousness, and use of a nasogastric tube. Subjects who had taken antituberculosis drugs for more than 3 days were excluded. Gastric lavage was performed in the morning after an overnight fast. Specimens were examined for direct smear, culture, and rapid molecular testing. Demographic, clinical, chest X-ray, and laboratory data were also recorded. During the study period, 31 subjects were available. The positivity rates for microbiological tests were 19.3%, 41.9%, and 48.4% for smear, culture, and rapid molecular testing, respectively. All positive smears were confirmed by either culture or rapid molecular testing. Gastric lavage can be considered a tool for improving extraneural TB diagnosis in unconscious patients.

## 1. Introduction

Tuberculosis (TB) is caused by *Mycobacterium tuberculosis* (*Mtb*). The World Health Organization’s report shows approximately 6.3 million new cases of TB every year, and Indonesia is the second country worldwide with the highest estimated incident cases of TB infection per year [1,2]. This condition is related to poverty, poor hygiene, malnutrition, and corticosteroid use and in the last decades was boosted by HIV coinfection [2,3].

*Mtb* initially infects the lung but can spread to other organs, such as meninges, bone, and kidney, with TB meningitis comprising around 1%–2% of the total TB infection. TB meningitis is the most severe extrapulmonary TB infection with high mortality and morbidity rate. TB meningitis is also the most common central nervous system infection in Hasan Sadikin Hospital, with a high mortality (more than 50%) [3,4,5].

A definite diagnosis of TB meningitis is challenging because of the paucibacillary nature of cerebrospinal fluid (CSF). For practical reasons, scoring systems are developed to weigh up the probability of the diagnosis of TB meningitis. One of the components of scoring systems is evidence of *Mtb* infection in the extracranial compartment, such as the lung [6]. Based on a previous cohort, 40% of TB meningitis patients have chest X-rays consistent with pulmonary TB, but a definite diagnosis of pulmonary TB is hard to establish due to hindrance in collecting sputum since 70%–85% of TB meningitis patients are admitted to the hospital with decreased consciousness [3,4,7].

It is assumed that unconscious TB meningitis patients swallow their sputum, so gastric lavage can be performed to obtain specimen for mycobacteriological examination and therefore can replace sputum collection in unconscious patients. As a matter of fact, gastric lavage is commonly performed on pediatric patients for the same purpose [8,9]. A mycobacteriological test of gastric aspirate is assumed to be an alternative way to improve the definite diagnosis of pulmonary TB in unconscious TB meningitis patients. This study aimed to increase the diagnosis certainty of pulmonary TB by performing microbiological examinations on gastric aspirate in unconscious TB meningitis patients.

## 2. Materials and Methods

### 2.1. Patients’ Characteristics and Study Design

A prospective study was conducted from January to March 2019 at Hasan Sadikin Hospital Bandung. Patients aged 18 to 60 years old with clinical TB meningitis and decreased level of consciousness and on nasogastric tube (NGT) were included in this study. Patients were excluded if they had taken antituberculosis drugs for more than 3 days. Lumbar puncture was performed on all the subjects included in the study to obtain CSF. All the subjects were also tested for HIV and had chest X-ray and head computed tomography (CT) as part of routine care. The patients received high-dose rifampicin (900 mg per day for 2 months), along with isoniazid, ethambutol, and pyrazinamide according to National TB Guidelines.

### 2.2. Gastric Lavage

Gastric lavage was performed on the subjects after an overnight fasting. Around 50 mL of normal saline was injected into the NGT. Then approximately 35 mL of gastric aspirate was drained from the NGT into a sterile falcon tube. Gastric aspirate was sent to the laboratory for further testing. Samples taken during weekends or holidays were stored in a refrigerator and processed on the next working days.

### 2.3. Sample Processing

Gastric aspirate was processed using 4% NaOH, then centrifuged at 3000 rotation per minute for 15 min. The sediment was collected for direct smear (Ziehl–Neelsen; ZN), liquid culture using microscopic observation of drug susceptibility (MODS), and rapid molecular testing using GeneXpert. Demographic data and results of the laboratory test, chest X-ray, and head CT were recorded.

### 2.4. Diagnostic Criteria

TB meningitis was suspected if the patients had clinical signs of meningitis (headache, fever, and neck stiffness with or without decreased level of consciousness). A probable diagnosis of TB meningitis was established if the CSF result revealed cells >10/mm [3] and low CSF:blood glucose ratio as described elsewhere [10]. A definite diagnosis of TB meningitis was obtained if the microbacteriological tests revealed a positive result in one diagnostic modality or more [10], while a definite diagnosis of pulmonary TB was made if either the culture or rapid molecular test or both revealed a positive result irrespective of the chest X-ray result [11].

### 2.5. Analysis and Statistics

Analysis and statistics were done by SPSS ver22.0. (IBM Corp, Armonk, NY, USA).

## 3. Results

### Patient Characteristics

**Figure 1:**
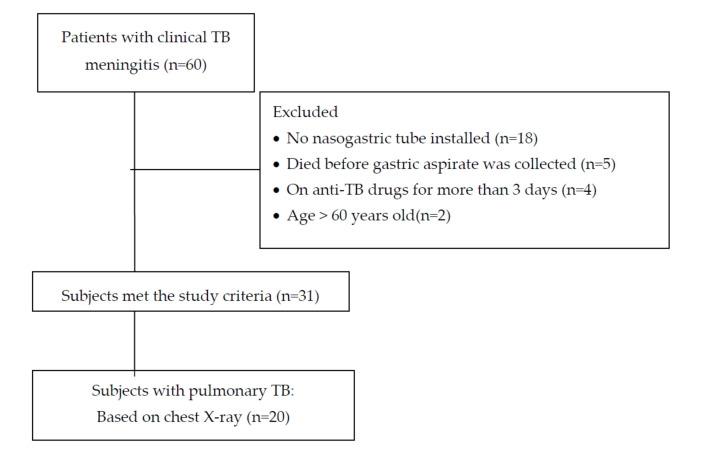
Subject flow.

Within the study period, there were 60 patients with clinical TB meningitis. Twenty-nine patients were excluded (18 patients were not on NGT due to being fully alert, 5 died before the gastric aspirate procedure was performed, 4 had already taken oral anti-TB for more than 3 days, and 2 were older than 60 years), leaving 31 subjects eligible for this study. Among these 31 subjects, 20 had suspected pulmonary TB based on chest X-rays, and *Mtb* was found in 17 subjects (Figure 1).

There was a slight female predominance in the study population (58%:42%), with a median age of 34 years. Around 10% of the study subjects had positive HIV serology, and 3 subjects (9.7%) had a history of pulmonary TB infection. The CSF profiles of the subjects were consistent with TB meningitis (i.e., increased white cell number with mononuclear cell predominance, high protein level, and low CSF:blood glucose ratio). Hyponatremia was common among the study subjects, with a median blood sodium level of 129 mEq/dL. Thirty of the 31 study subjects had grade 2 TB meningitis, 1 had grade 3 TB meningitis, and consistent with the inclusion criteria, there were no study subjects who had grade 1 TB meningitis.

Microbiological test results revealed that 17 (54.8%) had a positive result for rapid molecular testing, while 21 (67.5%) were culture positive, and 8 (25.8%) were direct staining positive. Positive composite mycobacterial test results (i.e., having positive result in one or more of the three mycobacteriological modalities performed) were obtained in 22 (71.0%) subjects.

Mycobacteriological examination of the specimens from gastric aspiration showed that 6 (19.4%) subjects had positive direct smear, whereas culture and rapid molecular testing were positive in 13 (41.9%) and 15 (48.4%) subjects, respectively. Definite pulmonary TB infection was found in 17 subjects (54.8%), based on composite mycobacterial testing. Twenty subjects (65.5%) had chest X-ray consistent with pulmonary TB, in which 14 (45.2%) subjects showed nonmiliary-type and 6 (19.4%) miliary-type pulmonary TB (Table 1). Half of the subjects with nonmiliary pulmonary TB and 5 of the 6 subjects with military TB had a positive microbiology result from gastric aspiration. Among the remaining 11 subjects with normal chest X-ray, 5 (45.4%) had a positive microbiology result. Further, of the 9 (29.0%) subjects without a definite diagnosis of TB meningitis, 5 (55.5%) had a positive microbiological result from gastric aspiration.

Five of the 6 subjects with miliary TB had microbacterial confirmation of TB infection, while only half of the subjects with nonmiliary-type pulmonary TB had confirmation. Interestingly, 5 of the 11 subjects whose chest X-ray showed no TB had a positive microbiology result (Table 2).

## 4. Discussion

The result of this study showed that mycobacterial confirmation from gastric lavage of unconscious TB meningitis subjects is quite satisfying, with 17 of the 31 subjects revealing a positive mycobacterial result, in which subjects with miliary TB based on chest X-ray had higher mycobacterial confirmation as compared with those with another chest X-ray abnormality (5/6 vs. 7/14). Further, subjects without chest X-ray abnormality still had quite a big proportion of mycobacterial confirmation (i.e., 5 out of 11), reinforcing the established standpoint that the diagnosis of pulmonary TB cannot be based on chest X-ray appearance only.

The positivity rates for the microbiological modalities were 48.4%, 42.0%, and 19.4% for rapid molecular testing, culture, and direct staining, respectively. These results are different from previous reports from studies using gastric aspirate, such as those from Baghaei (positivity rate for rapid molecular testing, 87%; culture, 85.7%; and direct staining, 66.7%) and Aslam (positivity rate for rapid molecular testing, 82.8%; culture, 67.8%; and direct staining, 61%) [12,13]. This discrepancy may be related to different study populations from that of this study. The subjects of this study were unconscious TB meningitis patients with or without known history of pulmonary TB, while the subjects of the above-mentioned studies were conscious patients with high suspicion of pulmonary TB based on history, clinical signs, and chest X-ray [12,13].

The positivity rate of rapid molecular testing in this study was a bit higher than that for sputum testing of suspected pulmonary TB patients from the general population in China (48.4% vs. 45.9%) [14]. A study in India also showed rapid molecular testing superiority over culture and direct staining using specimens from bronchoalveolar lavage and sputum [15]. Direct staining has the lowest positivity rate (19.4%), and positive direct staining is always confirmed by positive culture and/or positive rapid molecular testing. This may lead to a conclusion that direct staining is not necessary for examination in patients when rapid molecular testing or culture is available. All in all, performing TB tests from gastric aspirate can be considered to confirm pulmonary TB in unconscious subjects.

With regard to the HIV-infected subjects, 2 of the 3 subjects in this study had negative direct staining but positive culture and rapid molecular testing. A similar result was shown in a study conducted in Brazil, in which 53 of 78 HIV-infected subjects (68.0%) with pulmonary TB had negative direct staining but positive culture and/or rapid molecular testing [16]. A negative result of direct staining with a positive culture result is commonly found in pulmonary TB subjects coinfected with HIV [17].

Since a definite diagnosis of TB meningitis is difficult to attain, many clinical scoring systems have been developed to consider the possibility of certain patients having the disease, with extraneural TB diagnosis being one of the common parts of these scoring systems. One of the most commonly used scoring systems is a scoring from an international collaborative task force on tuberculous meningitis [6], which is usually referred to as Marais score. In this scoring system, extraneural TB adds up to the score. In this study, a definite diagnosis of pulmonary TB was achieved in 60% of the subjects with chest X-ray results consistent with TB infection (7 of the 14 subjects with nonmiliary pulmonary TB and 5 of the 6 subjects with military TB). This condition increases the score. Additionally, a definite diagnosis of pulmonary TB was obtained from 5 of the 9 subjects without a definite diagnosis of TB meningitis. Additional evidence of extraneural TB obtained from gastric aspiration results also adds to the score, thereby increasing the probability of having TB meningitis in this group of patients.

These results show that a microbiological test of gastric aspirate can help confirm pulmonary TB on unconscious TB meningitis patients. Another method for collecting samples for microbacteriological confirmation in cases of pulmonary TB with sputum smear negative for AFB is bronchial/bronchoalveolar lavage (BAL). In a study conducted in India, rapid molecular testing detected *Mtb* in 31% of all the patients, while culture at 6 weeks was positive in 22% [18]. In comparison with bronchoalveolar lavage, specimen sampling in gastric lavage is somewhat easier. The gastric lavage procedure is less invasive and presumably will not inflict additional harm to patients. Considering the result of this study, this gastric lavage procedure can be recommended to be undergone by unconscious patients, especially TB meningitis patients.

This study has several limitations. Clinically, we cannot be certain that the *Mtb* identified from gastric lavage really originated from swallowed sputum. Additionally, the protocol for TB culture of specimens from gastric lavage needs to be improved since the contamination rate is still high.

## 5. Conclusions

Microbiological testing of gastric aspirate improves the diagnosis of pulmonary TB in unconscious adults with TB meningitis. Compared with other microbiological modalities, rapid molecular testing is superior. Positive direct staining is always accompanied by positive rapid molecular testing, culture, or both.

## Figures and Tables

**Table 1:** Characteristics of subjects (*n* = 31).

**General Characteristics**	
Age in years—median (IQR)	34 (25–43)
Sex	
Male	13 (41.9)
Female	18 (58.1)
History of pulmonary TB	3 (9.7)
Positive HIV serology	3 (9.7)
**Laboratory examination**	
*Blood test*	
Serum sodium (mEq/L)—median (IQR)	129 (123–137)
Blood glucose (mg/dL)—median (IQR)	117 (94–128)
*CSF*	
Protein (mg/dL)—median (IQR)	191.7 (136–327)
Cell count (cell/mL)—median (IQR)	71 (6–195)
% lymphocyte—median (IQR)	81 (56–96)
% glucose ratio CSF:serum—median (IQR)	16.9 (10.4–35.8)
*Microbiology of gastric aspirate*	
Positive direct staining	6 (19.4)
Positive culture	13 (42.0)
Positive rapid molecular testing	15 (48.4)
**Radiology**	
*Chest X-rays*	
Not pulmonary TB	11 (35.5)
Abnormal:	
-Nonmiliary pulmonary TB	14 (45.2)
-Miliary pulmonary TB	6 (19.4)
*Head CT*	
Communicating hydrocephalus	10 (32.3)
Noncommunicating hydrocephalus	2 (6.45)
**Diagnosis**	
*Grade of TB meningitis* *	
Grade 2	30 (96.8)
Grade 3	1 (3.2)
*Tuberculous meningitis*	
Definite	22 (71.0)
Probable	4 (12.9)
Possible	5 (16.1)
*Pulmonary TB*	
Definite	17 (54.8%)

* Grade 1 TB meningitis was excluded, TB—tuberculosis, CSF—cerebrospinal fluid, IQR—interquartile range; All numbers are presented as *n*(%) unless stated otherwise.

**Table 2:** Comparison of chest X-ray and microbiology results.

Chest X-rays	Mycobacteriology Result *	
	Positive	Negative
No pulmonary TB (*n* = 11)	5	6
Nonmiliary pulmonary TB (*n* = 14)	7	7
Miliary pulmonary TB (*n* = 6)	5	1

* Positive mycobacteriology result was defined as having one or more positive results from the three mycobacteriological modalities used in this study.
